# Host Defense Mechanism-Based Rational Design of Live Vaccine

**DOI:** 10.1371/journal.pone.0075043

**Published:** 2013-10-02

**Authors:** Yo Han Jang, Young Ho Byun, Kwang-Hee Lee, Eun-Sook Park, Yun Ha Lee, Yoon-Jae Lee, Jinhee Lee, Kyun-Hwan Kim, Baik Lin Seong

**Affiliations:** 1 Department of Biotechnology, College of Life Science and Biotechnology, Yonsei University, Seoul, South Korea; 2 Department of Pharmacology, IBST, School of Medicine, Konkuk University, Seoul, South Korea; 3 Translational Research Center for Protein Function Control, Yonsei University, Seoul, South Korea; Mount Sinai School of Medicine, United States of America

## Abstract

Live attenuated vaccine (LAV), mimicking natural infection, provides an excellent protection against microbial infection. The development of LAV, however, still remains highly empirical and the rational design of clinically useful LAV is scarcely available. Apoptosis and caspase activation are general host antiviral responses in virus-infected cells. Utilizing these tightly regulated host defense mechanisms, we present a novel apoptosis-triggered attenuation of viral virulence as a rational design of live attenuated vaccine with desired levels of safety, efficacy, and productivity. Mutant influenza viruses carrying caspase recognition motifs in viral NP and the interferon-antagonist NS1 proteins were highly attenuated both in vitro and in vivo by caspase-mediated cleavage of those proteins in infected cells. Both viral replication and interferon-resistance were substantially reduced, resulting in a marked attenuation of virulence of the virus. Despite pronounced attenuation, the viruses demonstrated high growth phenotype in embryonated eggs at lower temperature, ensuring its productivity. A single dose vaccination with the mutant virus elicited high levels of systemic and mucosal antibody responses and provided complete protection against both homologous and heterologous lethal challenges in mouse model. While providing a practical means to generate seasonal or pandemic influenza live vaccines, the sensitization of viral proteins to pathogen-triggered apoptotic signals presents a potentially universal, mechanism-based rational design of live vaccines against many viral infections.

## Introduction

Vaccine remains the most effective means for controlling microbial infection and has greatly contributed to the control of infectious diseases [1]. With a view to developing live attenuated viral vaccines, attenuation has been achieved by repeated passages either in non-human cell lines [2] or in sub-optimal temperatures [3], [4], to accumulate random mutations that render the virus non-pathogenic in human hosts. A variety of genetic methods of attenuation have been recently advocated as alternatives to the classic methodology [5]–[7]. Attenuation of virulence is often achieved at the expense of the efficacy and the growth of the vaccine strain, and therefore, the development of clinically useful LAV still remains a highly empirical process, requiring a proper balance among safety, efficacy, and productivity.

Apoptosis and caspase activation are general host defense mechanisms against many viral infections to prevent the spread of the viruses by killing the infected cells [8], [9]. Viruses, however, modulate the apoptotic machinery to survive the wholesale destructive process and support their replication cycle [10]–[13]. A representative example is the active degradation of the cellular proapoptotic p53 protein by the human papillomavirus E6 protein and adenovirus E1B-55K protein [14], [15]. In addition, several viruses target the caspase activation process as well. The viral serpin CrmA protein encoded by most poxviruses inhibits caspase-1 and caspase-8 activation, blocking or delaying the cell death [16], [17]. More strikingly, there have been many convincing evidence that influenza viruses have evolved not only to avoid the apoptosis-mediated viral elimination but also to exploit and even activate it for their successful lifecycle. It was reported, for instance, that influenza viral proteins NS1 and PB1-F2 acted as apoptosis promoters [18], [19] and that caspase-3 activation was essential for efficient influenza virus replication [20]. Apparently, influenza NP and M2 are subjected to caspase-dependent cleavage during the viral infection, only to accomplish successful viral lifecycle [21]–[23], suggesting that influenza viruses outwit and exploit the host defense mechanism in favor of viral replication.

Given such an intimate relationship between the influenza virus and caspases, it is reasonable to predict that the introduction of artificial caspase cleavage site(s) into proper place in the influenza viral proteins would lead to degradation of the target proteins and sensitize the virus to apoptotic signals eventually resulting in the attenuation of virulence. Considering that the influenza virus, like many other virus infections, induces caspase-dependent apoptosis of infected cells [24]–[28], we designed a novel type of attenuation of the influenza virus which employs the caspases as the cellular scavengers of viral proteins. Using a reverse genetics system [29], we generated mutant influenza A viruses carrying caspase cleavage motifs in the viral nucleoprotein (NP) and nonstructural protein 1 (NS1). NP and NS1 proteins are crucially required for viral replication and antagonizing host-induced interferon responses, respectively [30], [31]. The mutant viruses were highly attenuated both in vitro and in vivo and induced strong immune responses with a single vaccination, providing complete protection against lethal challenges. Notably, the mutant viruses still demonstrated high growth ability in embryonated chicken eggs and hence were suitable for mass production. Extending popular mechanism-based drug design [32], [33], this may offer a novel and potentially general principle for designing live attenuated vaccines.

## Materials and Methods

### Ethics statement

This study was carried out in strict accordance with the recommendations of the Korean Food and Drug Administration (KFDA) guidelines. Protocols were reviewed and approved by the Institutional Animal Care and Use Committee (IACUC) of the Yonsei Laboratory Animal Research Center (YLARC) (Permit number: 2012-0094), and all the efforts were made to minimize suffering of mice.

### Animal cells

293T, A549, HeLa, and MDCK cells were cultured in minimum essential media (Hyclone Laboratories), supplemented with 10% FBS (Hyclone Laboratories). Primary chicken embryonic fibroblasts were isolated from 6-day-old egg and were cultured in Dulbecco's modified eagle medium (DMEM) (Hyclone Laboratories).

### PCR-based site-directed mutagenesis of influenza viral genes

PCR was used to introduce desired mutations into wild type influenza viral proteins. Using pHW2000 vectors each containing the wild type influenza gene segment 5 (NP) and 8 (NS) as template [29], PCR was performed with the primers including Asp-Glu-Val-Asp (DEVD) or Asp-Glu-Val-Ala (DEVA) amino acid sequences, by which the wild type amino acid sequences at position 431 to 434 in NP protein and 101 to 104 in NS1 protein were replaced by the DEVD or DEVA sequences. The mutated viral genes were then ligated into the pHW2000 vector and the mutant viruses were rescued by the reverse genetic procedure [29].

### Generation of recombinant influenza viruses by reverse genetics

293T cells were co-transfected with a mixture of eight plasmids each encoding a cDNA for the segment 1 to 8 of influenza virus (300 ng per each), plus Lipofectamine™ (Invitrogen) as recommended by the manufacturer. 48 hours (h) after the transfection, supernatants were harvested and examined for the presence of virus by plaque assay on MDCK cells. To obtain stock virus, initial supernatant was inoculated into 11-day-old embryonated chicken eggs. 72 h after the inoculation, allantoic fluid from the egg was harvested and viral titer was determined by plaque assay on MDCK cells. Reassortant DM-C:H5N1 virus was generated by combining the hemagglutinin (HA) and neuraminidase (NA) segments of A/Indonesia/5/2005 (H5N1) virus with other internal six genes from DM-C virus as a backbone. To ensure the safety of the DM-C:H5N1 reassortant virus, polybasic cleavage site (PQRESRRKKRG) of the HA segment of A/Indonesia/5/2005 (H5N1) was replaced by monobasic cleavage site (PQREKRG).

### Antibodies for western blot

Anti-NP or NS1 western blots were performed using polyclonal antibodies to NP or NS1 of A/WSN/33 (H1N1) virus, obtained from rabbits (LabFrontier) immunized with purified NP or NS1 proteins. Anti-caspase 3 western blot was performed using monoclonal rabbit antibody that can detect both full-length and cleaved fragments (Cell Signaling Technology, #9665). Anti-β-actin western blot was performed using polyclonal rabbit antibody (Abcam, #ab8227). As a secondary antibody, we used goat anti-rabbit IgG monoclonal antibody conjugated with horseradish peroxidase (Sigma, # SAB3700852).

### Serial passages of DM-C virus

The genetic stability of DM-C virus was examined by ten consecutive passages in eggs, MDCK cells, and A549 cells. For passages in eggs, ten eggs were inoculated with 100 plaque forming units (PFU) of viruses from previous passage. 72 h after inoculation, allantoic fluid from each egg was harvested and quantified by hemagglutinin assay using chicken red blood cells. Among these, the virus with the highest titer was selected and its PFU titer was determined on MDCK cells for the next passage. For serial passages in MDCK cells and A549 cells, 10^3^ PFU of viruses from the previous passage were grown on the cells for 48 h at 37°C, and then the supernatant was harvested and assayed for viral titers for the next passage. After ten passages, we sequenced RT-PCR products of NP and NS1 from plaque-purified viruses.

### Antibody analysis

Mice blood samples were taken by retro-orbital bleeding method and clotted at 4°C overnight to collect sera samples. We detected sera IgG antibodies and mucosal IgA antibodies by ELISA. Plates were coated with 10^5^ PFU of virus per well at 4°C overnight. After blocking and washing, plates were incubated with two-fold dilutions of sera or mucosal fluids for 1 h at room temperature (RT). After washing, the plates were incubated with HRP-conjugated secondary goat anti-mouse IgG antibody (Bethyl, # A90-116P) or IgA antibody (Bethyl, #A90-103P) for 1 h at RT. After washing, the plates were incubated with TMB substrate solution (BD Biosciences) for 30 min at RT in the dark. The colorimetric reaction was stopped by adding 2N H_2_SO_4_ solution and the absorbance was measured at 450 nm on ELISA reader. For hemagglutinin inhibition (HI) assay, sera were pre-treated with receptor destroying enzyme at 37°C overnight and then heated at 56°C for 30 min. 25 ul of two-fold dilutions of the sera were incubated with 4 HAU of influenza virus at 37°C for 1 h. 50 ul of 1% cRBC were added and incubated at 4°C for 1 h. The HI antibody titer was calculated as the reciprocal of the highest dilution that completely inhibited hemagglutination. Virus-neutralizing (NT) antibody titers were determined in sera pre-treated by heat of 56°C for 30 min. Two-fold dilutions of the sera were incubated with 100 PFU of influenza viruses at 37°C for 1 h and subsequently transferred to monolayers of MDCK cells for plaque assay. The NT antibody titer was calculated as the reciprocal of the highest dilution that yielded 50% inhibition of plaques compared to the control.

### Animal infection

For animal vaccinations and challenges, 6-week-old female BABL/c mice (OrientBio) were anesthetized with a mixture of ketamine and xylazine (1 and 0.2 mg per mouse, respectively) prior to intranasal infection with 50 ul of virus suspension. Pathogenicity of mutant viruses was studied with six to eight mice per group. After infection, their body weight and mortality were monitored daily over a period of two weeks.

### Preparation of mice tissue samples for viral titration

To collect the lung homogenates, nasal turbinates, and BALF, mice were anesthetized by a mixture of ketamine and xylazine and sacrificed by cervical dislocation to minimize suffering. The whole lung was removed from the sacrificed mouse and homogenized with electric homogenizer in the presence of 1 ml PBS. The homogenates were then centrifuged at 12,000 rpm for 10 min to remove the cell debris, and the clarified supernatants were transferred to new tubes and frozen at −80°C until analysis. Mucosal samples were collected as described previously [34]. Briefly, the nasal turbinates were collected by lavaging mouse nostrils repetitively with 200 µl PBS. To collect BALF, a catheter was inserted into the trachea, which was repetitively lavaged with 1 ml PBS. The nasal turbinates and BALF were clarified by centrifugation at 12,000 rpm for 10 min, and the supernatants were transferred to new tubes and frozen at −80°C until analysis.

### Quantitative real-time PCR

Total RNA was isolated from MDCK cells using TRIzol reagent (Life technologies) according to manufacturer's protocol. PCR was performed in 20 ul reactions with specific detection primer pair for canine IFN-β (sense: 5′- CCA GTT CCA GAA GGA GGA CA-3′; antisense: 5′- TGT CCC AGG TGA AGT TTT CC-3′). The mRNA level of IFN-β was assayed using the Thermal Cycler Dice Real Time System (Takara) and double strand specific dye SYBR Green system (Takara). The PCR conditions and cycles were as follows: initial reverse transcription 5 min at 42°C followed by 40 cycles of PCR reaction step: DNA denaturation step 95°C for 5 sec, followed by annealing and extension step 60°C for 30 sec. Relative quantitative evaluation was performed by the comparative ΔΔCt method. The ΔCt value of each gene obtained in mock-infected cells was used as calibrator, after normalization to endogenous control GAPDH gene (sense: 5′- TCG GAG TCA ACG GAT TTG GCC G-3′; antisense: 5′- GAC CCT CTT GGC CCC GCC T-3′). The results are expressed as an n-fold difference relative to calibrator (RQ = 2^−ΔΔCt^).

### GFP reporter gene transcription system

A cassette carrying a GFP reporter gene flanked by noncoding regions derived from influenza virus segment eight was inserted between RNA polymerase I promoter and terminator in pHH21 vector [35]. Transfection of the recombinant plasmid into 293T cells leads to the transcription of the reporter gene by RNA polymerase I and generates influenza vRNA-like negative sense RNA. When the plasmid is co-transfected with other plasmids expressing functional influenza polymerase complexes including PA, PB1, PB2, and NP proteins, the negative sense RNA serves as template for mRNA synthesis by the polymerase complexes, consequently resulting in translation into GFP protein.

### Multiplex cytokine ELISA

The concentrations of IL-1β, IL-6, and TNF-α in mouse BALF were determined using multiplex ELISA using MILIIPLEX kit (MILLIPORE).

### Virus plaque assay

All viral titers in the study were expressed as plaque forming units (PFU) calculated in plaque assay. Ten-fold dilutions of virus samples were absorbed into the well of confluent MDCK cells, on rocker at RT for 45 min. After the removal of the solution, cells were washed with PBS and agarose/DMEM overlay solution was added to each well. After the overlay turned solid, the plates were incubated in humidified incubator at 37°C and 5% CO_2_ until plaques were formed. To visualize the plaques, the cells were fixed by 4% formaldehyde and stained by crystal violet solution.

### Statistical analysis

All the values in vitro and in ovo experiments are expressed as the mean of at least three independent experiments, and the values in vivo experiments were the mean of each cohort. Error bars indicate the standard deviation (SD). Statistical analysis was performed using the unpaired Student t-test.

## Results

### Generation of mutant influenza A viruses carrying caspase cleavage motifs

**Figure 1:**
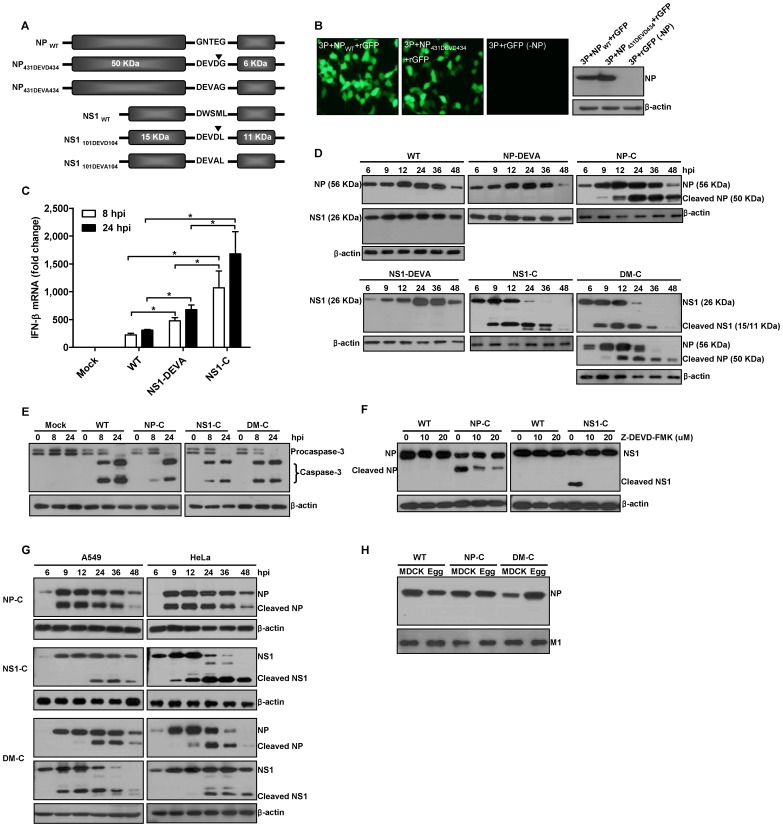
Generation of mutant influenza A viruses. (A) Schematic representations of mutant NP and NS1 proteins carrying DEVD or DEVA, on indicated position. ▾ indicates predicted caspase cleavage site. Each cleavage mutant virus, NP-C or NS1-C, carries mutant protein NP_431DEVD434_ or NS1_101DEVD104_, respectively, and double mutant virus, DM-C, carries both NP_431DEVD434_ and NS1_101DEVD104_. At the same position in NP or NS1 protein, DEVA sequences were introduced to create non-cleavage mutant virus, NP-DEVA or NS1-DEVA, respectively. (B) Transcription activity and expression of NP_431DEVD434_. 293T cells were transfected with plasmids encoding PA, PB1, PB2, with NP_WT_, NP_431DEVD434_, or without NP (-NP), together with GFP reporter plasmid. Fluorescence was observed at 24 h after the transfection. Cells lysates were subjected to anti-NP western blot. (C) Real-time RT-PCR quantification of IFN-β mRNA in MDCK cells infected with 10 MOI of each virus. IFN-β gene level was normalized with GAPDH gene level and presented as fold changes relative to mock-infected cells (* p<0.05). (D) Cleavage of mutant NP and NS1 in virus-infected cells. MDCK cells were infected with 10 MOI of each virus and the cells were harvested at different time points for anti-NP and NS1 western blots. (E) Caspase activation upon infection with WT or mutant viruses. MDCK cells infected with 10 MOI of each virus were harvested at different time points for anti-caspase-3 western blot. (F) Inhibition of cleavage by caspase-3/7 inhibitor, Z-DEVD-FMK. MDCK cells were infected with 10 MOI of each virus and the inhibitor was added to the media. The cells were harvested at 24 hpi for anti-NP and NS1 western blots. (G) Cleavage of mutant NP and NS1 in human cell lines. A549 and HeLa cells infected with 10 MOI of NP-C, NS1-C, or DM-C were harvested at different time points for western blot. (H) The status of NP in newly produced progeny viral virions. 10^5^ PFU of virions of WT, NP-C, and DM-C grown in MDCK cells or eggs were subjected to anti-NP western blot. M1 proteins were used as internal controls for western blot with the virions.

We designed influenza viral NP and NS1 proteins as artificial substrates of caspases. The mutational positions were carefully selected based on the reported structures of NP [36], [37] and NS1 [38], [39] to minimize the structural perturbation of the target proteins and thereby to maintain the high growth property of the viruses in vaccine production hosts such as embryonated chicken eggs. To ensure efficient cleavage of viral proteins by caspases, the Asp-Glu-Val-Asp (DEVD) motif, a well-known preferred cleavage site for group II effector caspase-3 and caspase-7, was chosen [40]–[42]. Using the viral genes of A/Puerto Rico/8/34 (H1N1) virus, we generated three cleavage mutant viruses, NP-C, NS1-C, and DM-C, each carrying the DEVD motif in NP, NS1, and both, respectively (Fig. 1A). In addition, two non-cleavage mutants, NP-DEVA and NS1-DEVA, each carrying Asp-Glu-Val-Ala (DEVA) at the same position in each target protein were also generated to differentiate the attenuating effects of caspase-mediated cleavage from structural changes caused by the amino acid mutations. First, we examined the effect of amino acid mutations on the expression level and the activity of the mutated NP protein. As shown in the plasmids transfection of Pol I-GFP reporter with influenza polymerase complexes, the DEVD motif on NP did not lead to decrease in the expression level and transcriptional activity of the protein, as the NP_431DEVD434_ could transcribe the influenza viral RNA (vRNA)-like GFP reporter template into mRNA as efficiently as NP_WT_ did (Fig. 1B). In the context of viral replication, however, the well-known function of the NS1 protein as an interferon (IFN)-β antagonist appeared to be significantly affected by the amino acids mutation, permitting higher expression of IFN-β mRNA than WT virus did (Fig. 1C). Of note, NS1-C demonstrated more profound defect in the IFN-β antagonism than NS1-DEVA did, suggesting that the NS1 was subjected to degradation by caspases in NS1-C-infected cells (Fig. 1C). To confirm the caspase-mediated cleavage of viral proteins during viral infection, MDCK cells were infected with WT or mutant viruses, and the cell lysates were analyzed by western blots. While WT and the two non-cleavage mutant viruses, NP-DEVA and NS1-DEVA, produced only full-length NP and NS1 proteins, those proteins of cleavage mutant viruses, NP-C, NS1-C, and DM-C, were efficiently cleaved from 9 hours post-infection (hpi) (Fig. 1D). Consistently, at similar time points, procaspase-3 appeared to be converted into active form upon the viral infection (Fig. 1E). In the presence of Z-DEVD-FMK, a caspase 3/7 inhibitor [43], the cleavage of NP and NS1 was markedly decreased (Fig. 1F), confirming that the cleavage was indeed mediated by caspases. Also, in A549 and HeLa cell lines of human origin, the viral proteins carrying DEVD motif were cleaved during infection, with varying degrees of cleavage efficiency depending on the cell line, target protein, and infecting viruses (Fig. 1G). The results suggest that the present strategy is also feasible and could be extended to humans. To examine whether the cleaved fragments of NP protein could be packaged into new progeny viral particles, the status of NP proteins in WT and mutant virus virions produced in MDCK cells or eggs were analyzed by western blots. Only full-length NP proteins were detected in both NP-C and DM-C mutant virions (Fig. 1H), implying that the cleaved products in virus-infected cells were excluded from incorporation into new virion particles.

### Growth properties in vitro

**Figure 2:**
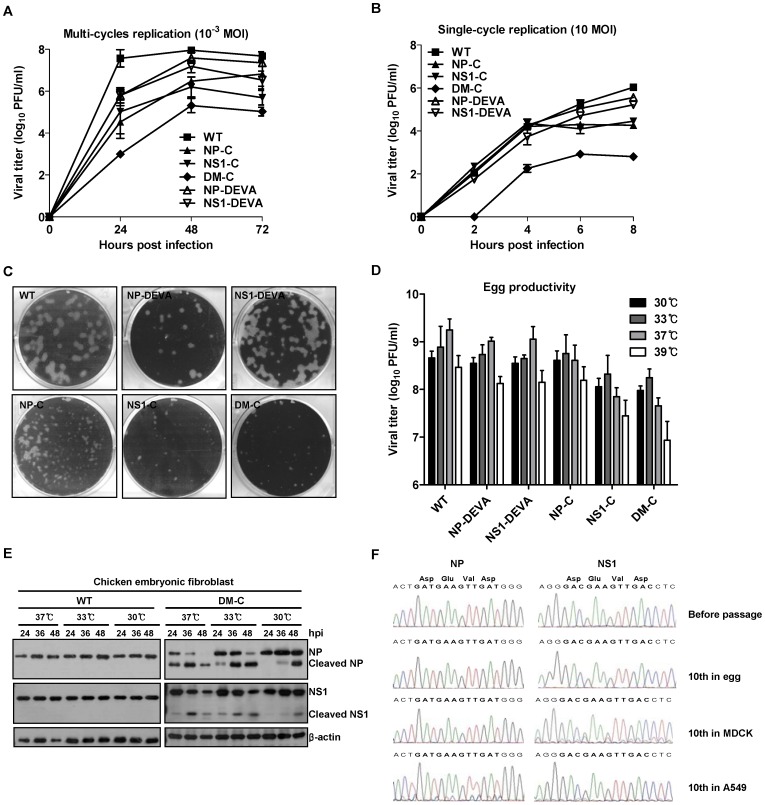
Growth properties and genetic stability of caspase-cleavage mutant viruses. (A) Multi-cycle replication of mutant viruses in MDCK cells. MDCK cells were infected with 10^−3^ MOI of each virus and the supernatants were harvested every 24 hpi. (B) Single-cycle replication of mutant viruses. MDCK cells were infected with 10 MOI of each virus and the supernatants were harvested every 2 hpi. Viral titers of the supernatants were determined by plaque assay. Data are the mean of three independent experiments. (C) Plaque-forming ability of mutant viruses. Viral plaque assay with WT and mutant viruses were performed on MDCK cells and the plaques were stained at five days after the assay. The representative images of crystal violet-stained plaques were shown. (D) Viral yields of mutant viruses in embryonated chicken eggs. Each egg was inoculated with 100 PFU of each virus and incubated at various temperatures. Four days later, the alantoic fluids were harvested for viral titrations. Data are the mean of at least 5 eggs. (E) Cleavage of mutant NP and NS1 in chicken embryonic fibroblasts (CEF). Primary CEF were infected with 1 MOI of WT virus or DM-C and incubated at various temperatures. The cells were harvested at different time points and the cell lysates were subjected to anti-NP and NS1 western blots. (F) Chromatograms showing nucleotide sequences encoding caspase cleavage motifs in NP and NS1 of plaque purified DM-C after ten passages in eggs, MDCK cells, and A549 cells.

To examine the correlation of caspase-mediated cleavage of viral proteins and the attenuation of viral virulence, we compared the growth ability between cleavage and non-cleavage mutant viruses in MDCK cells. In multi-cycle replication with the low initial infection titer of 10^−3^ multiplicity of infection (MOI), NP-C and NS1-C exhibited slower growth kinetics than WT virus, whereas each non-cleavage mutant virus showed intermediate growth ability between WT and cleavage mutant virus (Fig. 2A). DM-C carrying two independent cleavage motifs in NP and NS1 exhibited much reduced replication ability, showing that the attenuation effect could be enhanced by multiple introductions of the caspase cleavage motifs into the viral genome. The decreased growth of the non-cleavage mutant viruses compared to the WT virus suggests that both caspase-mediated cleavage and structural perturbation of target proteins contributed to the overall attenuation. Single-cycle replication with high initial infection titer of 10 MOI enabled us to specify the timing of the onset of growth restriction by caspase-mediated cleavage of viral proteins (Fig. 2B). While WT and non-cleavage mutant viruses which viral proteins are not cleaved by caspases continued to grow throughout 8 hpi, cleavage mutant viruses stopped the growth between 4 and 6 hpi, implying that NP and NS1 proteins started to be cleaved by caspases at those time points. The growth kinetics of each virus appeared to be well reflected in the plaque-forming ability; while non-cleavage mutant viruses, NP-DEVA and NS1-DEVA, produced comparable or only marginally smaller plaques compared to WT virus, all three cleavage mutant viruses produced much smaller plaques than WT and non-cleavage mutant viruses (Fig. 2C).

### Egg productivity and genetic stability

Besides the attenuated phenotype, a live influenza vaccine should maintain high growth properties in embryonated chicken eggs to be amenable to mass production, considering that the majority of influenza vaccines are currently being produced in eggs. To evaluate the growth properties of mutant viruses in eggs, we inoculated 100 PFU of WT or cleavage mutant viruses into each egg, with non-cleavage mutant viruses included as controls. Overall, the viral titers of the mutant viruses were lower than those attained by WT virus (Fig. 2D). However, there was apparent phenotypic distinction in the temperature profiles of the viral growth between the non-cleavage mutants and cleavage mutant viruses. While the non-cleavage mutant viruses, similar to the WT virus, showed optimal growth properties at 37°C, all three mutant viruses displayed the most robust growth at 33°C (Fig. 2D). Considering the attenuation mechanism involving enzyme activity of caspases, it is likely that the temperature at which viral replication occurs may affect the enzyme activity and the kinetics of cleavage of NP and NS1 in eggs. To test this hypothesis, chicken embryonic fibroblasts (CEF) isolated from 6-day-old egg were infected with WT virus or DM-C and incubated at various temperatures. As incubation temperatures dropped from 37°C to 30°C, the cleavage of NP and NS1 of DM-C virus were delayed for more than 12 hours in CEF (Fig. 2E). Thus, despite pronounced attenuation, DM-C maintains a robust growth (>10^8^ PFU/ml) in eggs in favor of lower temperature, most possibly by the decrease in the cleavage efficiency of caspases. The results suggest that it is possible to use egg-based mass production facilities at lower temperatures, as is the case for the cold-adapted live influenza vaccines [44], [45]. In addition, DM-C showed considerable genetic stability in its caspase cleavage motifs during repetitive replication cycles in various hosts. After ten consecutive passages in eggs, nucleotide sequences in viral RNA genome encoding the caspase cleavage motifs in NP and NS1 remained unchanged (Fig. 2F). After ten passages in MDCK cells, however, the chromatogram for the cleavage motifs in NS1 showed a low degree of heterogeneity, especially at the first base of the codon for Glu and Val, suggesting a minor population of Lys and Phe, respectively, at corresponding positions. Moreover, the genetic stability after serial passages in human A549 cell line (Fig. 2F) presents a safety net of a live attenuated influenza vaccine for human use.

### Attenuation in mouse model

**Figure 3:**
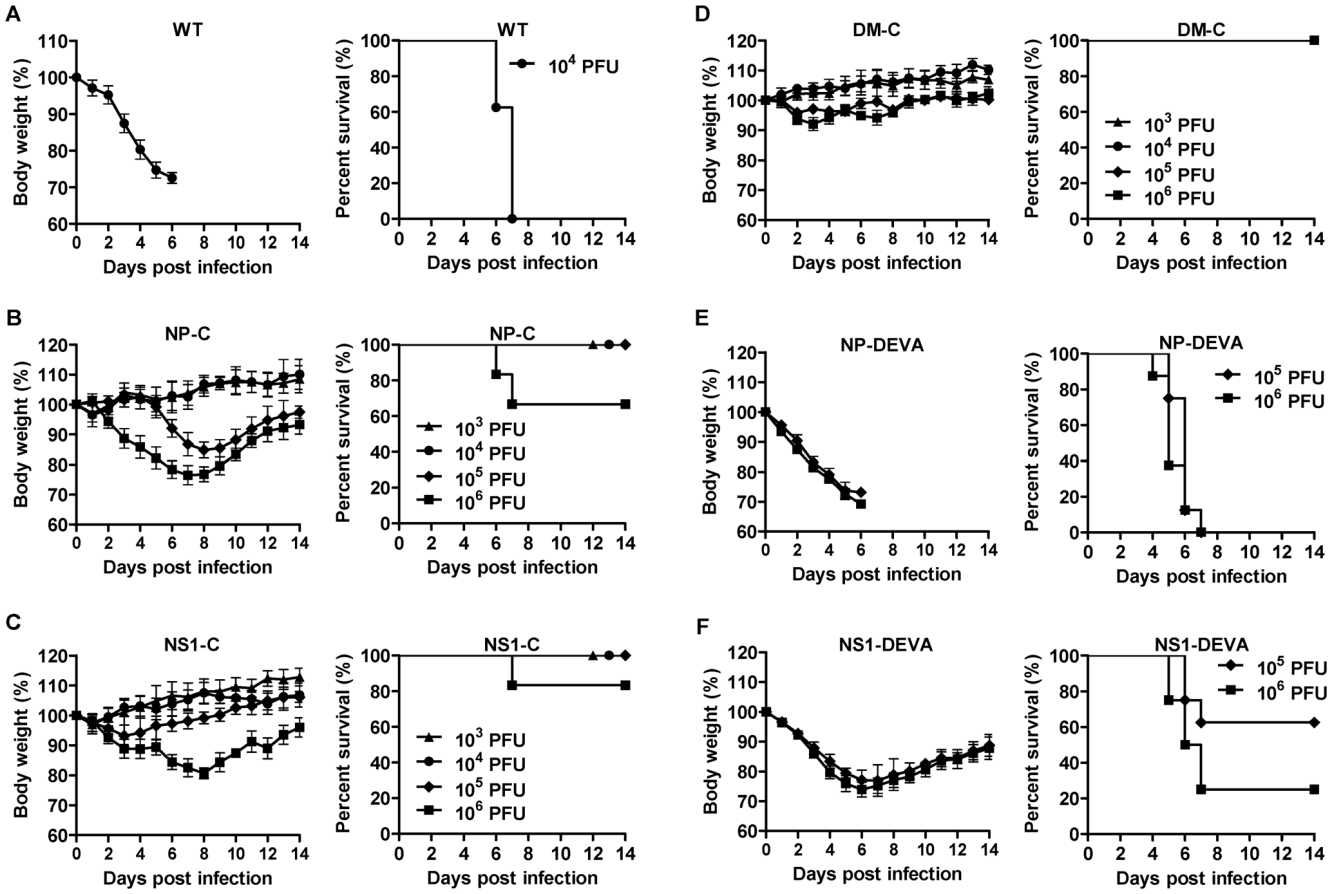
Attenuation of mutant viruses in mice. Seven to eight mice per group were intranasally infected with each virus and their morbidity and mortality were monitored daily for two weeks. (A–F) Time-course changes of body weights (left) and survival rates (right) of mice infected with various doses of WT virus (n = 8) (A), NP-C (n = 8) (B), NS1-C (n = 7) (C), DM-C (n = 8) (D), NP-DEVA (n = 8) (E), or NS1-DEVA (n = 8) (F). Data are the means of each cohort, and error bars indicate SD.

We then examined the virulence of WT and mutant viruses in mice. Mice were infected with 10^3^ PFU to 10^6^ PFU of each mutant virus, with 10^4^ PFU of WT virus included as a control. Infection with 10^4^ PFU of WT viruses was lethal, in which all mice lost their body weight rapidly and finally succumbed on 7 day post-infection (dpi) (Fig. 3A). Both NP-C and NS1-C, on the other hand, exhibited significantly reduced virulence compared to WT virus, and showed only partial lethality at the highest 10^6^ PFU infection dose (Figure. 3B,C). Thus, the mouse lethal dose 50 (MLD_50_) of the NP-C and NS1-C was increased by more than 1,000-fold compared with the parental WT virus. We failed to observe any lethality of DM-C, with only 10% loss of body weight at the highest infection dose (Fig. 3D), suggesting that the two independent attenuations are additive or synergistic. The level of attenuation correlates with and reflects the growth kinetics in cell culture (Fig. 2A,B). A parallel study with non-cleavage mutant viruses again enabled us to discriminate the attenuating effects of the caspase-mediated cleavage from structural changes of target proteins. Infections with 10^5^ PFU and 10^6^ PFU of NP-DEVA and NS1-DEVA were considerably lethal, leading to much greater morbidity and mortality than NP-C and NS1-C (Fig. 3E,F). NP-DEVA was completely lethal at 10^5^ PFU, whereas NP-C did not cause any lethality at the same dose (Fig. 3E). Likewise, NS1-DEVA exhibited much more morbidity and mortality than NS1-C (Fig. 3F). The results show that the caspase-dependent cleavage of target proteins contributes greatly to the observed attenuation. Although the amino acids change contributed in part to the attenuation of the viruses, the results confirm that the cleavage event of viral proteins is indispensable for the full expression of the desired level of attenuation.

### Analysis of attenuation of DM-C in mice

**Figure 4:**
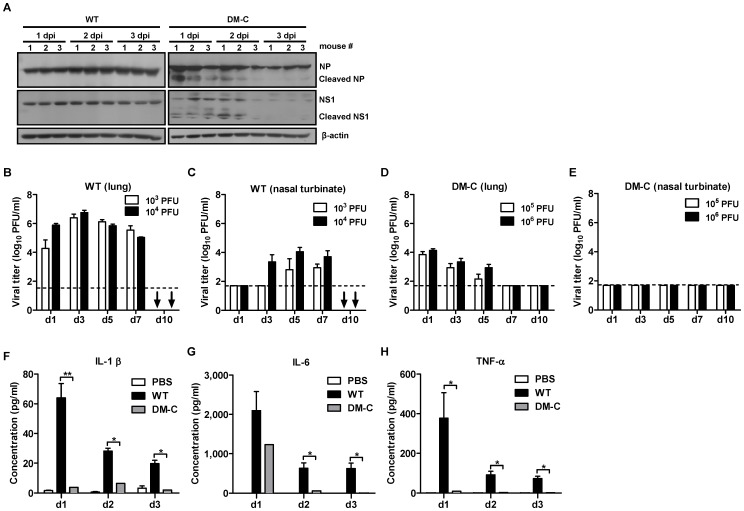
Analysis of attenuation phenotypes of DM-C. (A) The cleavage of mutant NP and NS1 in mice lungs. Three mice per groups were infected with 10^5^ PFU of WT virus or DM-C and the lungs were harvested daily. The lung homogenates were lysed and subjected to anti-NP and NS1 western blots. dpi, days post-infection. (B-E) Kinetics of viral replications in the respiratory tracts of mice infected with 10^3^ PFU or 10^4^ PFU of WT virus (B,C) or with 10^5^ PFU or 10^6^ PFU of DM-C virus (D,E). The mice were sacrificed at indicated days, and the lung homogenates and the nasal turbinates were prepared for viral titration by plaque assay. (↓) No mice left alive at 10 dpi following the WT virus infection. Dashed horizontal lines indicate the detection limits of each assay. (F–H) The expression levels of proinflammatory cytokines in mice infected with WT virus or DM-C. Three mice per groups were infected with 10^5^ PFU of WT virus or DM-C, and the BALF were collected from the mice daily after the infection. The concentrations of secreted IL-1β (F), IL-6 (G), and TNF-α (H) were estimated by multiplex ELISA (* P<0.05, ** P<0.01).

Exhibiting the most attenuated phenotype, DM-C was further examined for its attenuation in vivo. Western blot analysis showed the cleavage of mutant NP and NS1 as well as the time-dependent degradation of those proteins in DM-C-infected mouse lungs, while the WT proteins remained unaffected (Fig. 4A). Next, we examined the replication kinetics of DM-C in the respiratory tracts of infected mice. When mice were infected with 10^3^ PFU and 10^4^ PFU of WT virus, we could detect the persistently high levels of viruses in the lungs and nasal turbinates up to 7 dpi till death on 10 dpi (Fig. 4B,C). In contrast to WT virus, DM-C, even at higher infection doses of 10^5^ PFU and 10^6^ PFU, showed a great decrease in viral titers on 1 dpi in the lungs, and the titers were further decreased to below the detection limit on 7 dpi (Fig. 4D). Moreover, we failed to detect any remaining viruses in the nasal turbinates throughout the 10 days after infection (Fig. 4E). While inflammatory responses have several beneficial effects in combating influenza infection, the deregulation or excessive production of inflammatory cytokines in the lungs have been associated with immunopathogenesis of the infection, which become more pronounced in the influenza pandemics [46], [47]. To assess the expressions of inflammatory cytokines following infection with DM-C, we quantified the amount of representative proinflammatory cytokines, including tumor necrosis factor-alpha (TNF-α), interleukin-1β (IL-1β), and IL-6, in the bronchoalveolar lavage fluid (BALF) of infected mice. DM-C infection induced the secretion of IL-1β and TNF-α to only barely detectable levels. The level of IL-6 was approximately half that of WT virus on 1 dpi, which rapidly declined on 2 dpi (Fig. 4F–H). The results suggest that the impairment of viral replication and the cytokine production contribute collectively to highly attenuated properties of DM-C in animal model.

### Antibody responses and protection against lethal challenge

**Figure 5:**
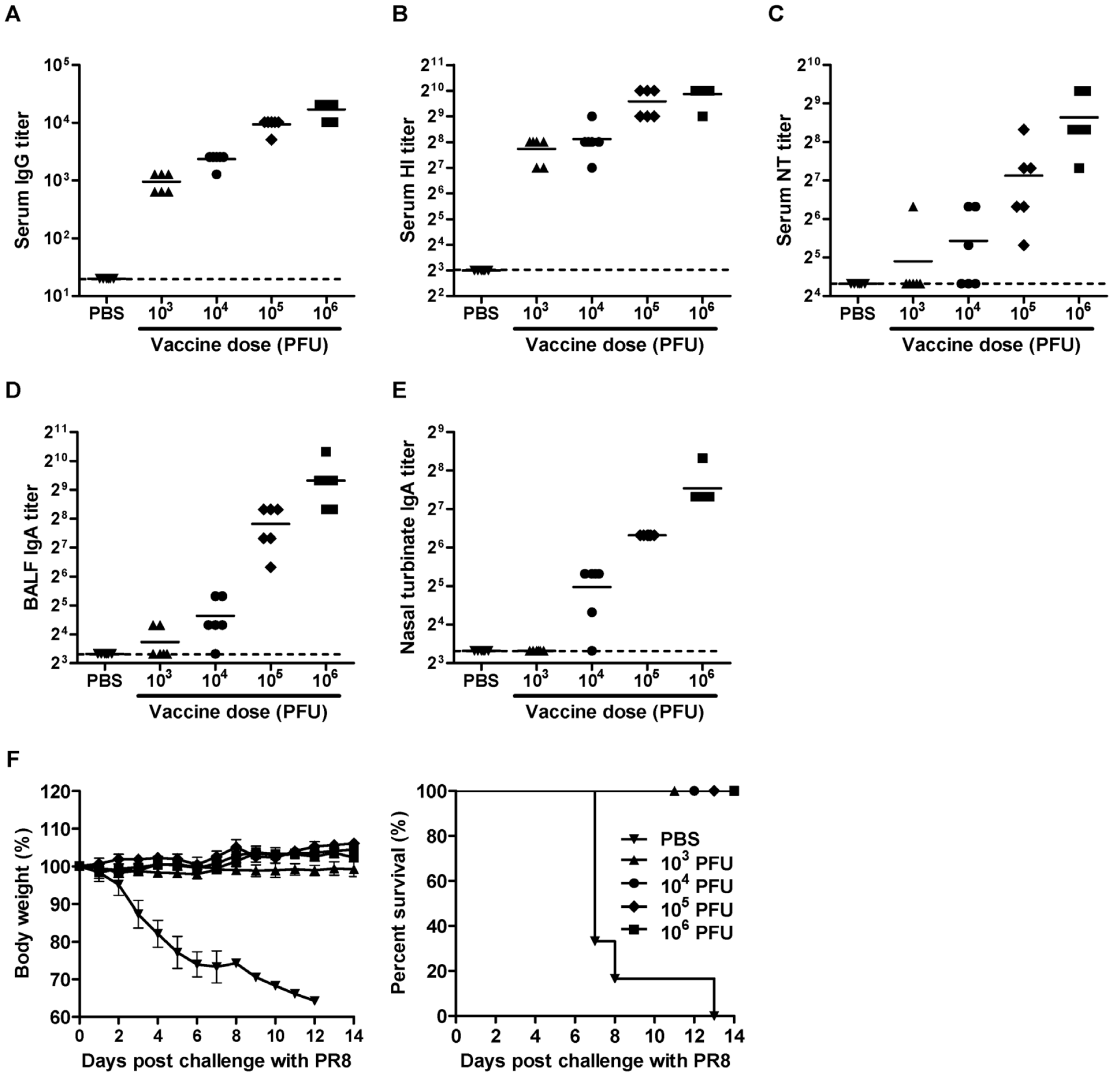
Antibody responses and protection against lethal challenge. (A–E) Antibody titers elicited by vaccination with DM-C. Six mice per groups were vaccinated with various doses of DM-C, or mock-infected with PBS as a control. Three weeks after the vaccinations, sera, BALF, and nasal turbinates were collected from the mice. PR8 virus-specific serum IgG (A), HI (B), and viral neutralizing (NT) antibody (C), as well as mucosal secretory IgA antibodies in BALF (D), and nasal turbinates (E) were quantified. Each value was plotted individually in the panel, with the mean of each cohort shown as a line. Dashed horizontal lines indicate the detection limits of each assay. (F) Protection against lethal challenge with WT virus. The vaccinated mice and PBS control groups were challenge with 10 MLD_50_ of WT viruses at four weeks after the vaccination. Their body weights (left) and survival rates (right) following the challenge were monitored daily.

To examine the immunogenicity and protective efficacy of DM-C, Mice were immunized with various doses of the virus. Upon a single vaccination, mice developed, in a dose-dependent manner, high titers of serum IgG antibodies, hemagglutinin-inhibitory (HI), and viral- neutralizing (NT) antibodies (Fig. 5A–C). Of note, even the lowest vaccine dose of 10^3^ PFU induced a mean HI antibody titer of 2×10^2^, which is approximately 5 times the conventionally defined level as protective in humans. In addition to systemic antibody responses, mucosal secretory IgA antibodies in the respiratory tracts were also effectively stimulated by the vaccination (Fig. 5D,E). To investigate the protective efficacy of the induced antibody responses, mice were challenge with 10 MLD_50_ of WT viruses four weeks after the vaccination. While non-vaccinated control mice exhibited rapid weight loss and 100% lethality after the challenge, all vaccinated groups survived and did not develop any morbidity even with the lowest vaccine dose of 10^3^ PFU, indicating the robustness of protection induced (Fig. 5F).

### Vaccine efficacy of DM-C:H5N1 reassortant virus

**Figure 6:**
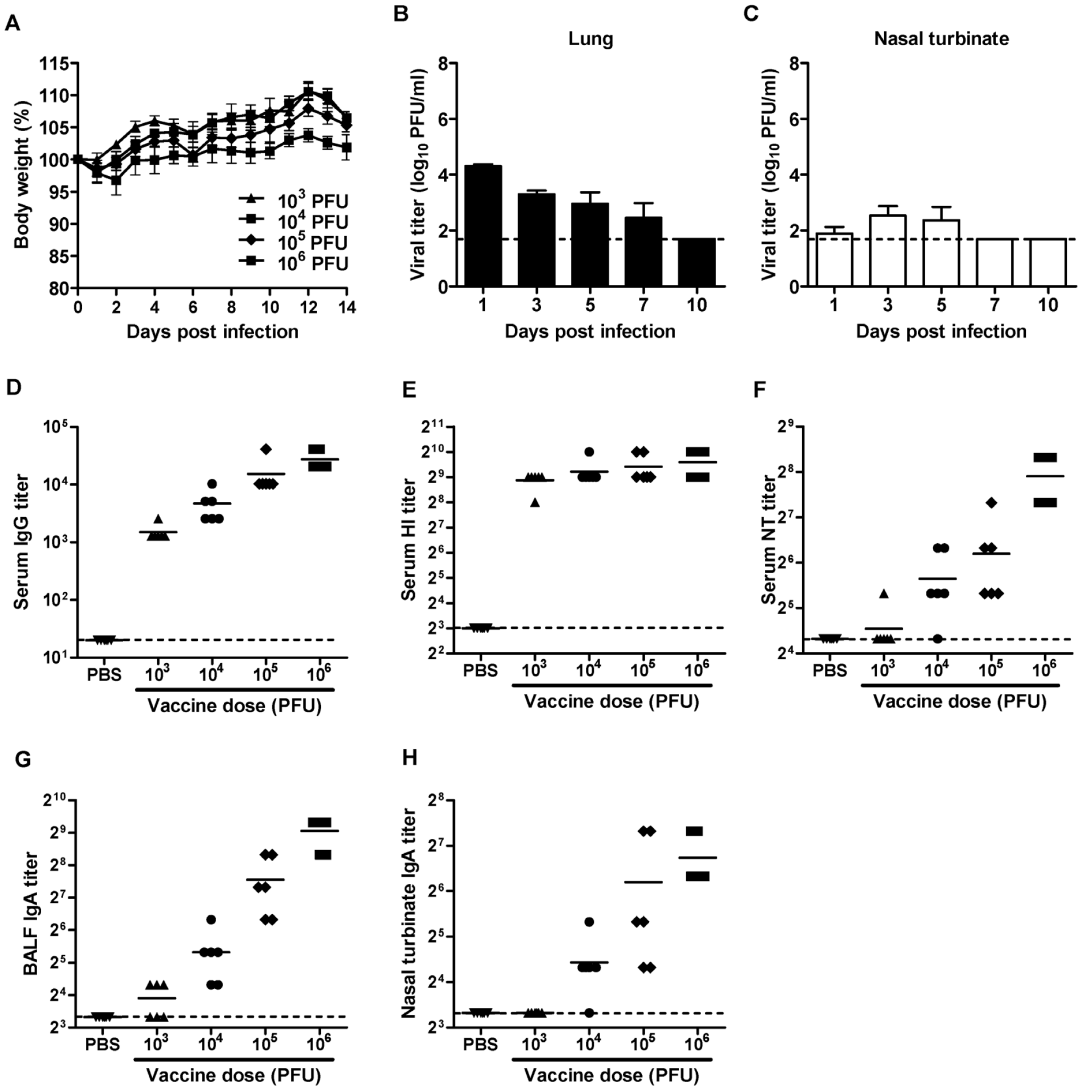
Attenuation and immunogenicity of reassortant H5N1 vaccine. (A) Body weight changes of the mice (n = 6 per group) infected with various doses of DM-C:H5N1 reassortant virus. (B,C) Replication of DM-C:H5N1 in the respiratory tracts in infected mice. Four mice per group were infected with 10^6^ PFU of DM-C:H5N1, and the lungs (B) and nasal turbinates (C) were harvested at different time points for viral titration. (D–H) Three weeks after the vaccination with DM-C:H5N1, the mice were examined for the induction of H5N1-specific serum IgG (D), HI (E), and NT antibodies (F), as well as mucosal secretory IgA antibodies in the BALF (G) and nasal turbinates (H).

**Figure 7:**
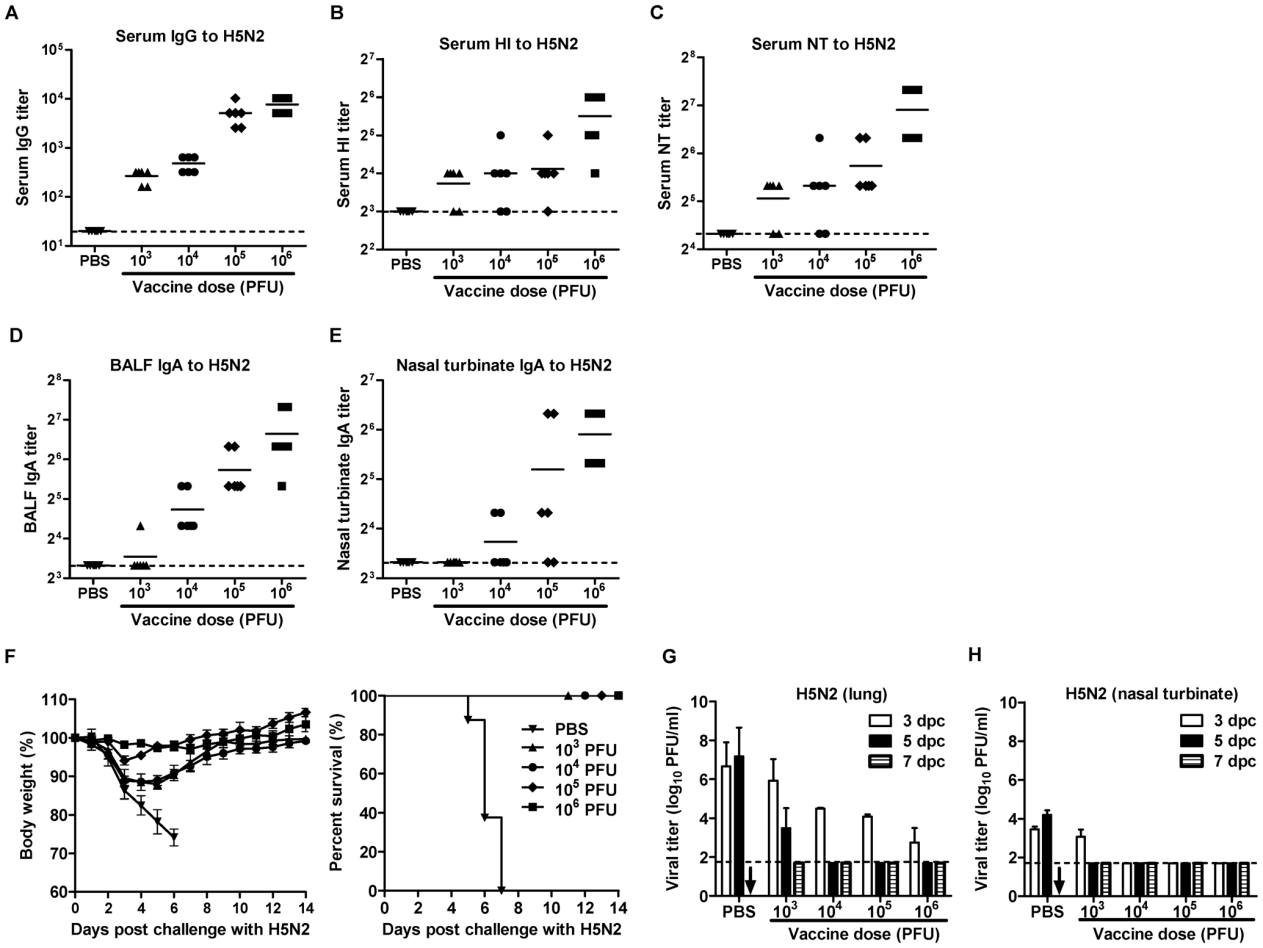
Protection of DM-C:H5N1 vaccine against H5N2 challenge. (A–E) Cross-reactive serum IgG (A), HI (B), NT antibody titers (C) and mucosal IgA antibody titers in the BALF (D) and nasal turbinates (E) to A/aquatic bird/Korea/w81/05 (H5N2) virus. (F) The vaccinated mice and PBS control mice were challenged with 10 MLD_50_ of the H5N2 virus, and their body weights changes (left) and survival rates (right) were monitored daily. (G,H) The H5N2 challenge virus clearance in mice vaccinated with DM-C:H5N1. Four mice per group were vaccinated with various doses of DM-C:H5N1, or mock-infected with PBS, and four weeks later the mice were challenged with 10 MLD_50_ of the H5N2 virus. At different time points, the lungs (G) and the nasal turbinates (H) were harvested for viral titration. (↓) No mice left alive at 7 dpi following H5N2 virus challenge.

To test the versatility of this strategy for designing a LAV, we generated a reassortant virus, DM-C:H5N1, consisting of two surface genes, HA and NA, from highly pathogenic avian H5N1 influenza strain (A/Indonesia/5/2005), and the six internal genes from DM-C. DM-C:H5N1 did not developed any morbidity in infected mice (Fig. 6A), and the virus was rapidly cleared in the respiratory tracts of infected mice (Fig. 6B,C), implying that the reassortant virus was also highly attenuated. Despite attenuation, DM-C:H5N1 virus remained highly immunogenic, inducing high levels of serum IgG antibody, HI antibody, and NT antibodies (Fig. 6D–F), as well as mucosal secretory IgA antibodies in the BALF and nasal turbinates (Fig. 6G,H). The protective efficacy of DM-C:H5N1 was tested using a mouse-adapted strain of A/aquatic bird/Korea/w81/05 (H5N2) [48] as a challenge virus. As expected, the cross-reactivity of sera and mucosal antibodies to the heterosubtypic H5N2 virus were much lower than to the homologous H5N1 virus (Fig. 7A–E). Nevertheless, the vaccination with DM-C:H5N1 completely protected the mice from lethal challenge with 10 MLD_50_ of H5N2 virus, without causing any lethality even with the lowest 10^3^ PFU vaccine dose (Fig. 7F). Cross-protection efficiency was further supported by the clearance of the challenge virus in the respiratory tracts. In vaccinated mice, challenge viruses were completely cleared on 5 or 7 day post-challenge (dpc) in the lungs and nasal turbinates, whereas non-vaccinated mice suffered from persistent viral replications in those tissues and finally succumbed on 7 dpc (Fig. 7G,H).

## Discussion

Live attenuated vaccines closely mimic natural infection and, via specific antibody- and cell-mediated immune responses, usually provide excellent protection against infection. In this study, we presented a novel attenuation of viral virulence based on general host defense mechanisms. Satisfying all three pillars of live vaccine–attenuation of virulence, productivity, and protective efficacy–, the apoptosis-triggered post-translational cleavage could be applicable to all the encoded viral proteins, including internal as well as surface proteins. It is likely that the level of attenuation correlates with the number of target proteins amenable to caspase-mediated cleavage. Based on increasing knowledge on the relationships between structure and function of influenza viral proteins, it may be possible to make a judicious choice of position of cleavage motifs in order to render target proteins more susceptible to caspases while minimizing structural perturbation. One advantage of this approach is therefore that through the combination of positions or the number of viral proteins, attenuating effect can be finely tuned to attain optimal balance between the attenuation and the immunogenicity of a live vaccine.

Traditionally, cold-adaptation by repeated passages at low temperature proved successful for attenuating the virulence to develop safe and effective live influenza vaccines [3], [44], [49]. With the availability of reverse genetics of influenza virus [50], a variety of genetic methods of attenuation have been advanced as alternatives to the cold-adaptation, including the repressive modification of specific viral target such as NS1, HA, or M2 [51]–[53]. The design of clinically useful live influenza vaccine remains, however, a difficult task because the attenuation of virulence often compromises the vaccine growth in production hosts, thus limiting its practical use [54]. As a novel approach on attenuation of virulence at the post-translational level, the present strategy distinguishes itself from previous attenuation methods targeting transcription by microRNA [7] or translation by codon deoptimization [5] of viral genes. Previously, the designing principle LAV has been basically drawn from the infecting agents and their virulence factors. Rather than targeting individual viral proteins and their respective functions [51]–[53], the present strategy is based on the apoptosis mechanism, a ubiquitous and general host response against infections, and therefore could be extended to the majority of infectious agents. Furthermore, the present strategy could be coupled to any other preexisting attenuation tools to increase the safety level of a vaccine. Especially, preferred growth of the caspase-cleavage mutant viruses at lower temperature closely resembles cold-adapted influenza live attenuated vaccines [44], [45], and hence the combination of these two strategies would provide a practical way to greatly improve the safety of a live attenuated influenza vaccine, especially for high risk groups.

Consideration of general host responses upon infection, as represented by the apoptosis machinery in this report, would provide new attenuation strategies and much wider choice of target proteins, and therefore could be implemented for the rational design of LAV. In concert with the classic attenuation via repeated passage at non-human cells [2] or sub-optimal temperatures [3], [4], in which attenuation characters are accrued randomly, the present apoptosis-triggered attenuation would contribute and expedite a mechanism-based design of live vaccines with desired balance of safety, efficacy, and productivity.
